# Amphiphilic Styrene-Based Pyrene Derivatives: Tunable Aggregation Luminescence and Their Photo-Induced Dimerization Behavior

**DOI:** 10.3390/molecules30081719

**Published:** 2025-04-11

**Authors:** Junying Zhang, Xingwei Luo, Juan Qiu

**Affiliations:** Anti-Aging Cosmetics Shandong Engineering Research Center, School of Chemistry and Chemical Engineering, Qilu Normal University, Jinan 250200, China; qiujuan@qlnu.edu.cn

**Keywords:** aggregation-induced emission, pyrene, self-assembly, luminescent material, photochemical reaction

## Abstract

Since the discovery of the aggregation-induced emission (AIE) phenomenon, various stimuli-responsive materials have been rapidly developed. However, how to achieve the transition between aggregation-caused quenching (ACQ) and AIE through molecular design is an urgent problem to be solved. In this work, we synthesized and studied the aggregation luminescence behavior and photochromism of two different substituted pyrene ethylene derivatives, **1-H** and **1-CN**. Due to the different substituents attached to the ethylene unit, **1-H** exhibits ACQ luminescence behavior. When the substituent is a cyanide group, it exhibits AIE behavior. In addition, the ordered nanoparticles formed by self-assembly in aqueous solution exhibit interesting photo-induced cyclization behavior, which leads to fluorescence quenching under ultraviolet light irradiation (λ = 365 nm). Therefore, due to their amphiphilicity and photo-responsiveness, these compounds can be used as anticounterfeiting inks in information encryption. This work contributes new members to the family of amphiphilic photo-responsive materials and demonstrates their potential applications in optical information storage and multi-color luminescence.

## 1. Introduction

Aggregation-induced emission (AIE) materials have become a fascinating class of luminescent materials with unique optical properties [1,2,3,4,5]. The AIE phenomenon has enormous potential applications in biological probes, chemical sensors, and optoelectronic materials [6,7,8,9,10,11]. Since Tang et al. proposed the concept of AIE in 2001, it has received widespread attention and has since made significant progress [12]. In many traditional systems, luminescent materials typically emit strongly in dilute solutions but, when the molecules aggregate, they encounter varying degrees of aggregation-caused quenching (ACQ) effects. In contrast, non-emissive luminescent materials are induced to emit through the formation of aggregates in AIE systems. AIE and ACQ represent two contrasting photophysical phenomena governed by distinct molecular mechanisms. In AIE systems, non-emissive molecules in solution become highly luminescent upon aggregation due to restricted intramolecular motion (RIM), which suppresses nonradiative decay pathways such as rotation or vibration of aromatic rings. This mechanism is exemplified by tetraphenylethylene (TPE) derivatives, where the restriction of phenyl ring rotations in aggregates activates radiative transitions. In contrast, ACQ arises in conventional fluorophores (e.g., pyrene) due to detrimental intermolecular π-π stacking interactions in aggregated states, which promote exciton migration and nonradiative energy dissipation [13]. Therefore, AIEgens exhibit significant advantages over typical ACQ molecules, particularly in their aggregated and solid-state states [14,15]. However, materials that exhibit both AIE and ACQ effects and can emit light in both solution and aggregated states are rarely reported in the literature [16,17].

In recent years, there has been a new trend in the research of AIE materials, which combines the performance of AIE materials with ACQ materials to fill the gap between the two. The combination of AIE and ACQ effects is consistent with the goal of other strategies, which is to achieve precise control of luminescent behavior and provide many interesting properties. However, existing preparation methods often require high temperatures or special conditions, and the synthesis process is complex and costly, which limits their practical applications and dissemination. This solution and solid dual emitting luminescent material can completely fill the gap between ACQ and AIE materials, providing many interesting properties such as stimulus-responsive fluorescence and white light emission. Therefore, there is a great need to develop material systems that can emit light in both solution and aggregated states.

Typical AIEgens, such as hexaphenylsilyl (HPS) and tetraphenylethylene (TPE), have been extensively studied [18]. In solution, the dynamic rotation of the benzene ring in TPE serves as a relaxation channel for exciton dissipation. During the formation of aggregates, this movement is restricted due to restricted intramolecular rotation (RIR) and intermolecular π-π stacking interactions originating from highly distorted molecular conformations [19,20]. By immobilizing propeller-shaped AIE molecules with various functional groups, new AIEgens will form and exhibit many different characteristics. This design method also utilizes the emission characteristics of AIE active cores, providing novel physical properties and applications for the new system.

Pyrene, a typical four-membered ring conjugated structure, is a polycyclic aromatic hydrocarbon with important application potential [21,22,23,24]. Meanwhile, pyrene and its derivatives are molecules with excellent fluorescence properties, emitting strong blue fluorescence at low concentrations, while pyrene exhibits the properties of excimer emission at high concentrations or in the solid state, leading to a decrease or quenching of fluorescence intensity and accompanied by a red shift [25,26,27,28]. This unfavorable phenomenon limits the application of pyrene and its derivatives as efficient solid-state luminescent materials in many fields. Since the establishment of aggregation luminescence science, the aggregation luminescence behavior of pyrene-based materials has been widely studied. In order to address the ACQ phenomenon of pyrene aggregation, researchers utilized basic AIE mechanisms such as intramolecular motion restriction (RIM), excited-state intramolecular proton transfer (ESIPT), and twisted intramolecular charge transfer (TICT) to transform pyrene-based ACQ emitters into AIE emitters with excellent optical properties [29]. However, there are currently few reports on pyrene-based AIE materials that can emit light in solution and aggregated states through the design and regulation of molecular functional groups [30].

**Scheme 1:**
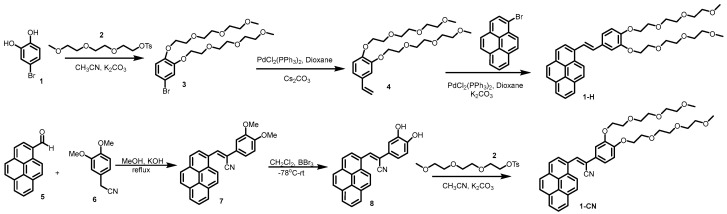
The synthetic route of luminescent compounds **1-H** and **1-CN**.

In this work, a novel class of pyrene-based amphiphilic luminescent materials was synthesized using a simple method. As shown in Scheme 1, when R is equal to H, compound **1-H** exhibits blue–green fluorescence in a well-dispersed state and gradually decreases in fluorescence intensity in an aggregated state. It is worth noting that, when R is equal to CN, molecule **1-CN** exhibits blue–green fluorescence in a well-dispersed state and, after increasing the viscosity of the molecules, the fluorescence intensity increases without a significant change in the emission wavelength. After adding water, the fluorescence emission peak of the **1-CN** aggregated state showed a significant red shift (96 nm) compared to the dispersed state, achieving precise luminescence control over a wide wavelength range. Compared with other reported pyrene-based organic luminescent materials, the compounds prepared in this work by adjusting the vinyl substituent groups still retain the inherent ACQ characteristics of pyrene-based materials and can also exhibit AIE effects at high concentrations or in aggregated states, enabling them to emit light in both solution and aggregated states. In addition, the introduction of unsaturated groups endows the material with interesting photo-responsive properties, enabling the conversion of different colors. The excellent performance of the novel aggregation-induced luminescent molecule **1-CN** has been further confirmed in the application of information encryption and multi-color visual sensing.

## 2. Results and Discussion

### 2.1. Synthesis Process and Characterization

The starting compounds (**1**, **2**, **3**, **7,** and **8**) used to synthesize the target dyes were known compounds in the literature and were obtained by the synthetic method found in the literature (Scheme 1) [31,32]. The whole preparation process, including the synthesis of precursors, consists of two steps: heck coupling reaction and Knoevenagel condensation reaction. The synthetic route is provided in Scheme 1. The polycyclic aromatic pyrene was introduced because pyrene molecules have tunable optical properties, modifiable functional groups and high photoluminescence efficiency. The ethylene-glycol-based hydrophilic block was attached to the fluorophore component, which had good biocompatibility in aqueous solution. Compound **1-H** amphiphilic fluorescent compound (Scheme 1) was prepared by Heck coupling reaction with bromopyrene unit and alkenyl derivatives modified with three ethylene glycol ether chains. Compound **7** powder was easily synthesized by reacting 1-Pyrene formaldehyde with the precursor under the action of basic catalyst, followed by simple purification. All products were characterized by ^1^H, ^13^C NMR, and mass spectrometry analysis (see ESI).

### 2.2. Photophysical Measurements

**Figure 1:**
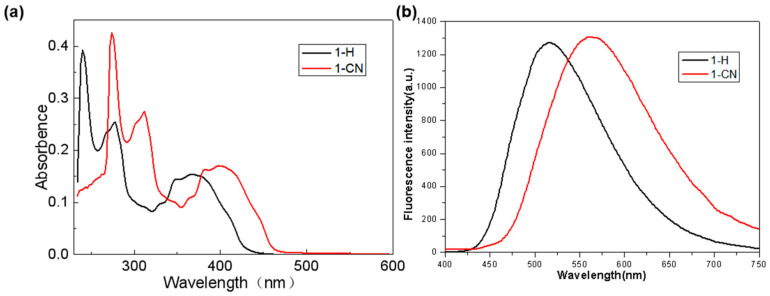
(**a**) Absorbance and (**b**) fluorescence emission spectra of **1-H** and **1-CN** in aqueous solution (1 × 10^−5^ M).

Two synthesized pyrene ethylene compounds are both fluorescent. The emission spectra and UV visible absorption spectra of **1-H** and **1-CN** were measured to investigate the optical properties of the prepared compounds in solution. Interestingly, the absorption and fluorescence properties of these two compounds are similar but show a certain wavelength shift. There are three characteristic absorption peaks in the UV visible absorption spectrum of compound **1-H** solution (Figure 1a). An absorption peak near 270 nm is attributed to the π→π* transition of the benzene ring. Another transition around 315 nm may be due to the π→π* red shift caused by the electron-donating effect of the methoxy group. The other is around 385 nm and the shoulder is around 410 nm, reflecting a larger conjugated system in the molecule. Figure 1 shows the emission spectra of compounds **1-H** and **1-CN**. The introduction of the cyanide group on the alkene bond causes a significant shift in the emission spectra of compound **1-CN** compared to **1-H** due to the electron-withdrawing effect. The emission peak in the fluorescence spectrum shifts from 514 nm to around 564 nm, with a red shift of 50 nm. When excited at 365 nm, the synthesized **1-H** aqueous solution exhibits bright cyan fluorescence at room temperature.

**Figure 2:**
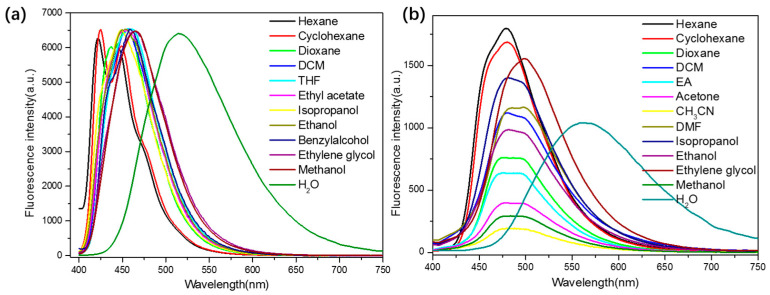
Normalized emission spectra of (**a**) **1-H** and (**b**) **1-CN** in different organic solvents and water (1 × 10^−5^ M).

We evaluated the emission spectra of the synthesized compounds **1-H** and **1-CN** in various solvents, including polar proton, nonproton, and dipolar nonproton solvents in 10^−5^ M solutions. The compound **1-H** exhibits dual emission peaks and shows significant solvation effects (Figure 2). The emission band that reaches its peak at 407 nm originates from the locally excited (LE) state and changes very little in different small polar solvents. However, as the solvent polarity increases (from n-hexane to methanol), another emission band shifts significantly from 444 nm to 464 nm, indicating intramolecular charge transfer emission. In water, this compound exhibits a wide orange yellow emission band (515 nm) due to the disturbance of the π- electron system caused by the formation of aggregates. It is worth noting that, in water, these compounds exhibit a significant emission wavelength shift (approximately 515 nm) and a decrease in intensity. These reduced intensities and wavelength shifts are attributed to the well-known ACQ phenomenon. In contrast, the fluorescence spectra of styrene-based pyrene compound **1-CN** showed significant changes in emission maximum and intensity (Figure 2b). At an excitation wavelength of 365 nm, the highest fluorescence of the compound was observed in low-polarity solvents (toluene, ether, dioxane, tetrahydrofuran, and ethyl acetate), with an emission maximum of approximately 478 nm (Figure 2b). The fluorescence spectrum in a more polar solvent shows a fluorescence band of approximately 500 nm, which is very consistent with the observed green color with the naked eye. The maximum fluorescence emission of compound **1-CN** shifts towards longer wavelengths in more polar organic solvents, as shown in the normalized emission spectra (Figure 2b). The significant color change from 500 nm to 564 nm (64 nm offset) when the solvent changes from ethylene glycol to water confirms the strong solvation discoloration behavior of **1-CN**. These properties are consistent with the lower electron-withdrawing ability of the H group in **1-H** compared to the CN group in **1-CN**. On the other hand, for the solution of **1-H** in all organic solvents, the same blue fluorescence with similar hue and intensity was observed, as shown in the normalized emission spectrum (Figure 2b). The slight changes in maximum emission wavelength and emission intensity indicate that the styrene-based pyrene probe without a push–pull system did not exhibit obvious solvation color change characteristics.

### 2.3. Aggregation Behavior Study

**Figure 3:**
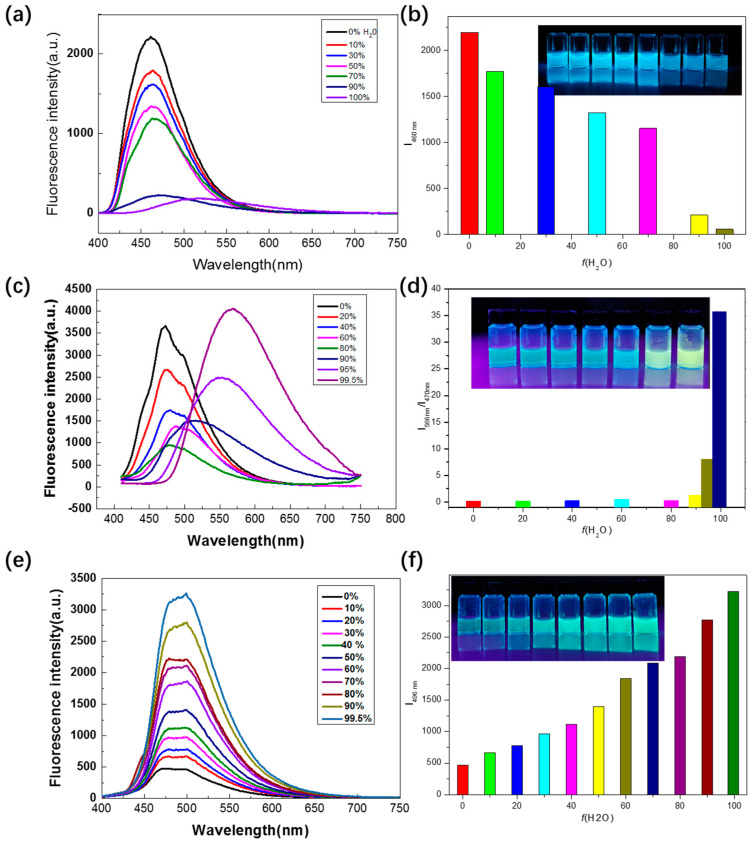
The emission spectra and the bar chart of the relationship between fluorescence intensity or ratio and the corresponding solvent system of (**a**,**b**) **1-H** and (**c**,**d**) **1-CN** in binary solvent mixtures of THF−water and the emission spectra of (**e**,**f**) **1-CN** in binary solvent mixtures of THF−ethylene glycol. The inset shows a distinct color change in the corresponding mixed solvents (1 × 10^−5^ M).

We investigated the relationship between the aggregation behavior and fluorescence of compounds **1-H** and **1-CN** and conducted fluorescence experiments in several common water-soluble organic solvents, including tetrahydrofuran (THF) and ethylene glycol. As shown in Figure 3a, compound **1-H** exhibits strong fluorescence in anhydrous THF. As expected, the fluorescence intensity gradually decreases with increasing water content in the solution. This means that dye **1-H** exhibits ACQ, and the origin of ACQ may be due to the π-π stacking of the pyrene ring (H-type stacking). A positive correlation was observed in the ratio plot of blank fluorescence to measured fluorescence intensity (F_0_/F) at a specified wavelength (F_460 nm_) and moisture content of 0–99.5% (*v*/*v*, Figure 3b). In the presence of water, fluorescence quenching is caused by the stability of the excited state, which leads to the promotion of nonradiative relaxation.

Next, we have attempted to convert ACQ-type **1-H** into AIE active substance **1-CN** by replacing the H on the double bond with CN. To our knowledge, cyanoethylene is a well-known AIE emitter. In cyanoethylene derivatives, the olefin stator is surrounded by aromatic groups and CN rotors, and the limitation of intramolecular rotation (RIR) process can explain its AIE effect. Many cyanoethylene derivatives with significant AIE properties have been reported. Therefore, we anticipate that compound **1-CN** will exhibit AIE characteristics. In order to explore the AIE activity of compound **1-CN**, their fluorescence emission behavior was tested in THF–H_2_O diluted mixed solvent systems with different volume water contents (f_w_, volume percentage of water in THF/H_2_O mixture). As shown in Figure 3c, in dilute THF solution, compound **1-CN** exhibits green fluorescence with a maximum wavelength of 470 nm. This phenomenon may be mainly attributed to the active intramolecular rotation of the compound, which is the relaxation channel of the excited state. When an appropriate amount of water is added to the THF solution, the emission peak weakens and shows a red shift due to the increase in solvent polarity, which is attributed to the ICT effect of compound **1-CN** with D-A structure in polar solvents. When f_w_ exceeds 90%, PL recovers and the PL intensity of compound **1-CN** begins to rapidly increase due to molecular aggregation. When f_w_ is 99.5%, the corresponding fluorescence intensity is increased by nearly six times compared to when f_w_ = 0. The bright yellow light emitted (centered at 566 nm) can be attributed to the formation of aggregates. The occurrence of this phenomenon can be explained by the decrease in solubility of **1-CN** in mixed solvents, which is due to the aggregation of molecules caused by the addition of water. Compared with the dispersed state, the interaction between molecules in the aggregated state is stronger, the internal torsional potential energy increases, and the rotational and vibrational motion within the molecules is suppressed. This leads to the release of excited-state energy through radiative transitions, thereby enhancing the fluorescence intensity in the mixed solution. In short, as the volume fraction of water increases, **1-CN** in the mixed solution first undergoes ACQ due to π-π stacking between molecules and then AIE due to stronger intermolecular interactions that limit intramolecular motion. In order to gain a deeper understanding of the AIE effect, the influence of solvent viscosity on fluorescence was studied. Compared with the weak fluorescence in low-viscosity solvents (THF), compound **1-CN** exhibits relatively strong fluorescence in ethylene glycol. In addition, the PL behavior of compound **1-CN** in THF/ethylene glycol mixtures containing different ethylene glycol components was also studied. As the proportion of ethylene glycol increases, the fluorescence intensity of compound **1-CN** gradually increases (Figure 3e). The above results indicate that the viscous medium inhibits intramolecular rotation, thereby suppressing nonradiative processes that lead to enhanced fluorescence emission. Quantitative photoluminescence quantum yield (PLQY) measurements (Table S1) reveal a stark contrast between **1-H** and **1-CN**: the former exhibits ACQ-driven quenching (PLQY: from12.5% to 3.1%), while the latter shows AIE-enhanced emission (PLQY: from 8.1% to 42.3%). This five-times enhancement for **1-CN** underscores the role of the -CN group in suppressing π-π stacking and activating RIM-dominated luminescence.

**Figure 4:**
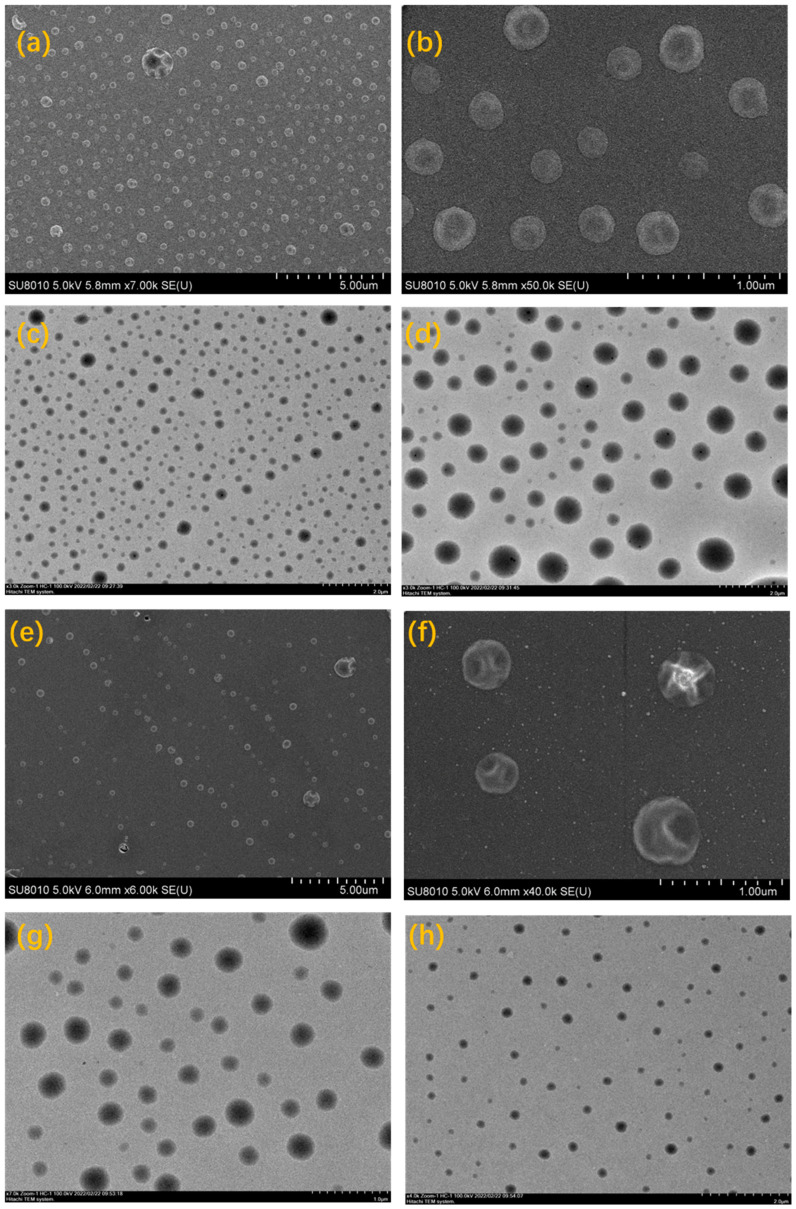
SEM image (**a**,**b**) and TEM image (**c**,**d**) of **1-H** prepared in aqueous solution. SEM image (**e**,**f**) and TEM image (**g**,**h**) of **1-CN** prepared in aqueous solution (1 × 10^−5^ M).

The presence of pyrene units and hydrophilic chains prompted us to study their self-assembly behavior and morphology through scanning electron microscopy (SEM) and transmission electron microscopy (TEM). As shown in Figure 4a,c, it can be clearly observed through SEM and TEM that compound **1-H** exhibits irregular spherical nanoparticles in aqueous solution. Meanwhile, the nanoparticles of compound **1-CN** exhibited similar morphology and size under the same conditions (Figure 4e,g). The additional SEM and TEM images of the two compounds are shown in Figure S11–S14. The successful formation of irregular spherical nanoparticles may be due to the hydrophobic pyrene units encapsulated in the core of the nanoparticles, while hydrophilic ethoxy ether chains are coated on their surface, giving them good water dispersibility. TEM confirmed the solid structure of self-assembled nanospheres. There is no significant difference in size distribution between the two substituent compounds. The substituent is neutral and accounts for a small proportion in the entire structure, with little impact on the balance between hydrophilicity and hydrophobicity. We performed DLS measurements on aqueous solutions of **1-H** and **1-CN** (1 × 10^−5^ M) to confirm the formation of aggregates and determine their hydrodynamic diameter. The results showed a hydrodynamic diameter of 120 nm−350 nm, consistent with the SEM/TEM observations of spherical nanoparticles (Figure S15).

### 2.4. Study on Photochemical Reaction

**Figure 5:**
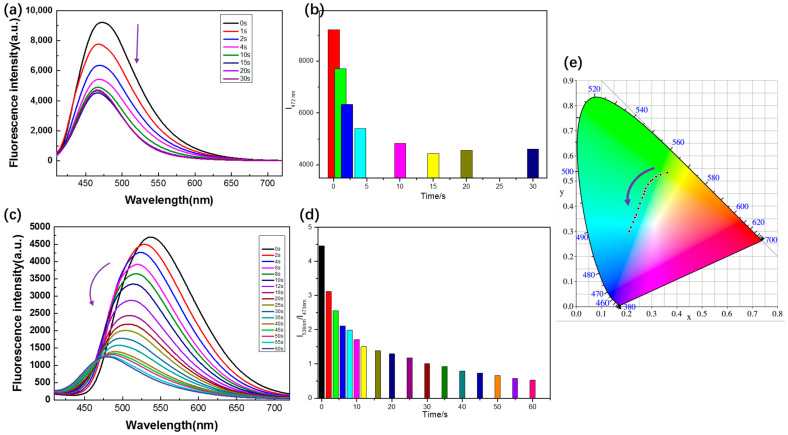
(**a**) The emission spectra of pyrene derivative **1-H** in aqueous solution over 30 s with exposure to 365 nm LED light and (**b**) the time dependence of the fluorescence intensity (I_472_ nm). (**c**) The emission spectra of pyrene derivative **1-CN** in aqueous solution over 60 s with exposure to 365 nm LED light and (**d**) the time dependence of the ratio of the fluorescence intensity (I_536_ nm/I_473 nm_). (**e**) CIE chromaticity diagram showing the time dependence of the (x, y) color co-ordinates of **1-CN**.

The excited-state dynamics of unsaturated bond compounds, including photo response, have long been a focus of research. Inspired by the cyclization of double-bond derivatives to cyclobutane derivatives after irradiation, we expect that the two compounds with double-bond units can also undergo structural transformation through cyclization [33,34,35]. Subsequently, a series of photophysical response behaviors were studied. Under the irradiation of ultraviolet light (λ = 365 nm, 20 W LED) in the air, the fluorescence intensity of compound **1-H** (1 × 10^−5^ M) at 472 nm gradually decreases with the prolongation of irradiation time in the aqueous solution, and the change in fluorescence emission cannot be detected after 30 s of irradiation (Figure 5a). Figure 5b illustrates the variation in fluorescence intensity of compound **1-H** with different irradiation times. As is well known, pyrene is a classic exciton complex molecule, so the decrease in fluorescence intensity may be due to the depletion of **1-H** and the subsequent production of pyrene excimer complexes. Similar phenomena were observed for compound **1-CN** in aqueous solution (Figure 5c). The quasi-molecular emission band at approximately 536 nm immediately decreases during irradiation and undergoes a blue shift with prolonged irradiation time, ultimately stabilizing at 437 nm (Figure 5c), corresponding to the emission band of pyrene’s excimer complex. After fluorescence quenching at 536 nm, the ratio of 536 nm fluorescence intensity to 437 nm fluorescence intensity changed from 4.46 to 1.70 after 10 s of irradiation, to 1.01 after 30 s of irradiation, and reached 0.63 after 60 s of irradiation, demonstrating high conversion efficiency. Figure 5e shows the changes in luminescent color at different exposure times in the International Commission on Illumination (CIE) 1931 [36] co-ordinate chromaticity diagram. From the CIE co-ordinates, it can be observed that the co-ordinates have shifted from (0.36, 0.53) to (0.21, 0.30), which is very consistent with the change in the emission spectrum, showing a color change from yellow green to blue.

**Figure 6:**
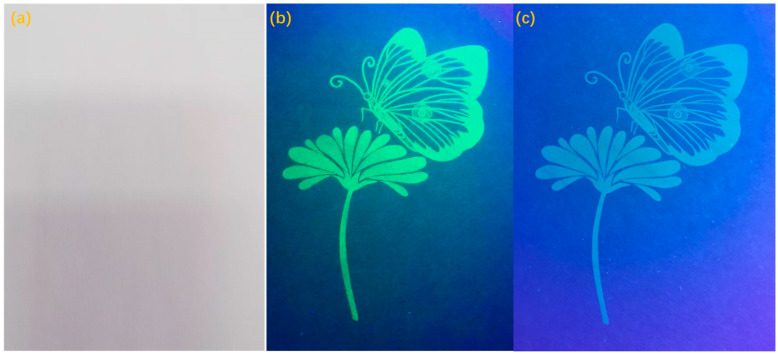
Photographs of fluorescent inkjet printing-based paper with **1-CN** (1 × 10^−5^ M) under (**a**) natural light and (**b**) ultraviolet light. (**c**) The color change after being exposed to 365 nm irradiation.

**Scheme 2:**
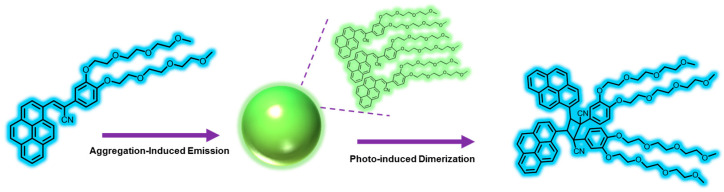
The schematic diagram of molecular aggregation and photo-cyclization.

Based on the analysis of the aggregation morphology of the previous compound, it is shown that the compound first forms micro-spherical structures through molecular aggregation, while achieving aggregation-induced luminescence enhancement. Subsequently, the luminescence color can be controlled and the luminescence intensity can be changed through the process of photo-induced cyclization (Scheme 2). Inkjet printing is an economically efficient, easily accessible, and high-throughput solution processing method used for depositing and incorporating functional materials onto paper substrates. We use inkjet printing technology and photo-induced luminescence discoloration to demonstrate the change of luminescence color because this is often useful in advanced information storage and security applications. To demonstrate the aggregation-induced luminescence and photochromic behavior of pyrene derivatives **1-CN**, we filled traditional inkjet printer ink cartridges with raw ink composed of a 0.01 mM amphiphilic fluorophore aqueous solution. Then, an image of the butterfly love flower pattern was printed on traditional nonfluorescent A4-sized printing paper (Figure 6). This pattern does not display under natural light; however, when visualized under 365 nm light, the inkjet-printed design exhibits bright green fluorescence emission, which visually approximates the color behavior of compound **1-CN** in aggregated solutions as previously described. Due to the photochemical reaction of pyrene-based compound **1-CN**, it turns blue within 60 s when exposed to LED light and its brightness is significantly lower than the previous green fluorescence, producing a pattern effect with sufficient contrast that can be observed with the naked eye. These experimental results indicate that this material has great potential as an invisible ink and color-changing luminescent material in the field of information security.

## 3. Materials and Methods

### 3.1. Materials and Instrumentation

Reagents and solvents were purchased from commercial sources (Macklin Biochemical Technology Co., Ltd., Shanghai, China). Compounds **3**, **7**, and **8** were prepared according to the reported method. ^1^H NMR and ^13^C NMR data spectra were recorded on a Bruker 400 MHz spectrometer (Bruker, Bremen, German) in CDCl_3_ at 298 K using TMS as the standard. Spin multiplicity is reported as singlet (s), doublet (d), and triplet (t), and coupling constants (J) are given in Hz or multiplet (m). Electrospray ionization (ESI) mass spectrometry analysis was performed using AgilentQ-TOF6510 (Santa Clara, CA, USA). High-resolution mass spectrometry (HRMS) was performed using ultra-performance liquid chromatography combined with quadrupole light time spectrometer. The UV-Vis absorption spectrum was measured by TU-1901 dual-beam UV-Vis spectrophotometer (Beijing Puxi General Instrument Co., Ltd., Beijing, China). The fluorescence spectra of the samples were measured with a Hitachi F-7000 fluorescence spectrophotometer (Hitachi Scientific Instruments (Beijing) Co., Ltd., Beijing, China) using a monochromatic Xe lamp as the excitation source. SEM images were obtained using s-4800 (Hitachi Ltd., Tokyo, Japan) at an accelerating voltage of 1.0 kV or 10.0 kV. Samples for transmission electron microscopy (TEM) measurements were prepared by dropping the solution on a copper grid, and the samples were examined by jem-1011 at an accelerating voltage of 100 v.

### 3.2. Synthesis

#### 3.2.1. Synthesis of Compound **4**

A mixture of vinylboronate pinacol ester (432.4 mg, 2.80 mmol), the corresponding aryl bromide 3 (900 mg, 1.87 mmol), Cs_2_CO_3_ (1.83 g, 5.60 mmol), and Pd_2_Cl_2_ (PPh_3_)_2_ (180 mg, 0.19 mmol) in dry dioxane (20.0 mL) was stirred at 95 °C for 5 h under a N_2_ atmosphere. Then, the reaction mixture was evaporated under vacuum. H_2_O (5 mL) was added to the residual mixture. The mixture was extracted with ethyl acetate (10 mL × 3), and the combined organic layer was dried over anhydrous Na_2_SO_4_, filtered, and the solvent was evaporated under vacuum. The residue was purified by silica gel chromatography using EtOAc/*n*-hexene as the eluent to afford the product **4** (680 mg, 85%). ^1^H NMR (400 MHz, CDCl_3_): δ 7.02 (s, 1H), 6.94 (d, *J* = 12.0 Hz,1H), 6.86 (d, *J* = 8.0 Hz, 1 H), 6.62 (m, 1H), 5.60 (m, *J* = 20.0 Hz, 1H), 5.14 (m, *J* = 12 Hz, 1H), 4.15–4.20 (m, 4H), 3.85–3.88 (m, 4H), 3.73–3.76 (m, 4H), 3.37 (s, 6H). ^13^C NMR (100 M Hz, CDCl_3_): δ 148.93, 148.91, 136.35, 132.13, 132.04, 131.95, 131.92, 131.42, 128.56, 128.44, 120.11, 114.50, 112.05, 112.30, 71.93, 70.83, 70.81, 70.70, 70.68, 70.55, 69.79, 69.73, 68.90, 68.85, 59.02. HR-ESI-MS: *m*/*z* calcd for C_22_H_37_O_8_: 429.2410 [M + H]^+^, found: 429.1965.

#### 3.2.2. Synthesis of Compound **1-H**

A single-necked flask was equipped with a stir bar charge with 1-bromopyrene (389 mg, 1.40 mmol), 4 (500 mg, 1.17 mmol), Pd_2_Cl_2_ (PPh_3_)_2_ (82 mg, 0.12 mmol), and K_2_CO_3_ (322 mg, 2.30 mmol). Then, the reaction flask was stirred at 100 °C for 12 h, and then the reaction mixture was cooled to room temperature. After removing the solvent under reduced pressure, the pure product was obtained by silica gel column chromatography (eluent: petroleum ether/ethyl acetate), 558 mg, 76%. ^1^H NMR (400 MHz, CDCl_3_): δ 8.49 (d, *J* = 12.0 Hz, 1H), 8.28 (d, *J* = 8.0 Hz, 1H), 8.11–8.18 (m, 3H), 7.97–8.05 (m, 4H), 7.65–7.69 (m, 1H), 7.43–7.48 (m, 2H), 7.26–7.30 (m, 1H), 6.96 (d, *J* = 8.0 Hz, 1H), 4.21–4.31 (m, 4H), 3.89–3.94 (m, 4H), 3.76–3.81 (m, 4H), 3.65–3.72 (m, 8H),3.53–3.57 (m, 4H),3.35–3.39 (m, 6H). ^13^C NMR (100 MHz, CDCl_3_) δ 138.55, 132.26, 131.29, 129.70, 128.61, 130.7, 128.59, 128.20, 127.46, 127.43, 126.31, 126.1, 126.15, 125.93, 124.98, 124.72, 124.56, 122.65, 119.27, 118.37, 114.66, 111.42, 109.09, 56.27, 56.12. HR-ESI-MS: *m*/*z* calcd for C_38_H_44_O_8_: 629.3036 [M + H]^+^, found: 629.3112.

#### 3.2.3. Synthesis of Compound **1-CN**

To a stirred suspension of compound 8 (190 mg, 0.52 mmol) and potassium carbonate (1.0 g, 7.23 mmol) in CH_3_CN (40 mL), a solution of ditosylate 2 (662.2 mg, 2.08 mmol) in CH_3_CN (25 mL) was added. The reaction mixture was stirred at 100 °C for 12 h, and the solvent was removed under reduced pressure. The resulting residue was triturated with chloroform (100 mL), and the organic phase was washed with water (50 mL × 3) and brine (50 mL) and dried over sodium sulfate. Upon removal of the solvent in vacuo, the crude product was subjected to column chromatography (EtOAc/hexane 1:4) to produce **1-CN** as a yellow gel (275.2 mg, 81%). ^1^H NMR (400 MHz, CDCl_3_): δ 8.61 (d, *J* = 8.0 Hz,1 H), 8.46 (s, 1H), 8.24–8.32 (m, 3H), 8.19 (d, 1H), 8.09–8.16 (m, 2H), 7.43–7.48 (m, 2H),8.03–8.07 (m, 1H), 7.80 (d, *J* = 8.0 Hz, 1H), 7.41 (s, 1H), 7.34 (d, *J* = 8.0 Hz,1H), 7.04 (d, *J* = 8.0 Hz, 1H), 4.25–4.3 (m, 4H), 3.91–3.94 (m, 4H), 3.77–3.80 (m, 4H),3.65–3.69 (m, 8H), 3.52–3.56 (m,4 H), 3.37−3.40 (m, 6H). ^13^ C NMR (100 MHz, CDCl_3_) δ 147.72, 146.33, 132.16, 130.79, 129.50, 129.30, 128.81, 127.74, 127.07, 126.66, 126.37, 125.58, 125.24, 124.26, 124.10, 123.93, 118.76, 118.33, 116.60, 115.24, 113.93. HR-ESI-MS: *m*/*z* calcd for C_39_H_44_NO_8_: 654.2989 [M + H]^+^, found: 654.3174.

## 4. Conclusions

In summary, we synthesized two amphiphilic pyrene-based derivatives, **1-H** and **1-CN**, which exhibit distinct AIE and ACQ behaviors. The cyanostilbene-functionalized **1-CN** achieves precise control over luminescence through solvent polarity-driven aggregation and photo-induced cyclization, enabling dynamic fluorescence switching from green to blue. Beyond its utility as a photoresponsive ink for anticounterfeiting, this system holds significant promise for advanced functional material applications. The time-dependent fluorescence quenching of **1-CN** under UV irradiation allows for time-gated decryption, where encrypted messages become readable only after specific irradiation durations. This aligns with emerging demands for dynamic anticounterfeiting in smart packaging and IoT devices. The irreversible erasure of fluorescence signals suggests potential for eco-friendly rewritable paper or smart tags, reducing waste in single-use security labels. Similar systems have been validated for sustainable temporary displays. The solvent-dependent AIE/ACQ transition could be harnessed for real-time polarity or viscosity sensing, such as in lipid droplet imaging or polymerization monitoring, leveraging the AIEgen’s environment-sensitive emission. These applications align with recent advances in AIE materials for biomedical imaging, wearable sensors, and energy-saving devices, positioning our platform as a versatile tool for next-generation functional materials.

## Data Availability

Data are contained within the article and Supplementary Materials.

## Supplementary Materials

The following supporting information can be downloaded at: https://www.mdpi.com/article/10.3390/molecules30081719/s1, Figures S1–S9: ^1^H NMR spectrum, ^13^C NMR spectrum, and HR-ESI-MS spectrum of compound **4**, **1-H**, and **1-CN**; Figure S10: The emission spectra of **1-CN** in binary solvent mixtures of THF–water; Figures S11–S14: SEM and TEM images of **1-H** and **1-CN** prepared in aqueous solution; Figure S15. The DLS data of **1-H** and **1-CN** prepared in aqueous solution; Table S1: The Photoluminescence Quantum Yield of compound **1-H** and **1-CN**.
