# Supplementary material for: Amphiphilic Styrene-Based Pyrene Derivatives: Tunable Aggregation Luminescence and Their Photo-Induced Dimerization Behavior
Source: Molecules. 2025 Apr 11;30(8):1719. doi: 10.3390/molecules30081719 (PMC12029862; doi:10.3390/molecules30081719)
Supplement: Supplementary file 1 [file molecules-30-01719-s001.zip › molecules-3509833-supplementary.pdf]

Amphiphilic Styrene-Based Pyrene Derivatives: Tunable Aggregation Luminescence and Their Photo-induced Dimerization Behavior

Junying Zhang *, Xingwei Luo* and Juan Qiu

Anti-aging Cosmetics Shandong Engineering Research Center, School of Chemistry and Chemical Engineering, Qilu Normal University, Jinan 250200, PR China

* Correspondence: jyzhang@qlnu.edu.cn (Z. J.); xluo@qlnu.edu.cn (L. X.)

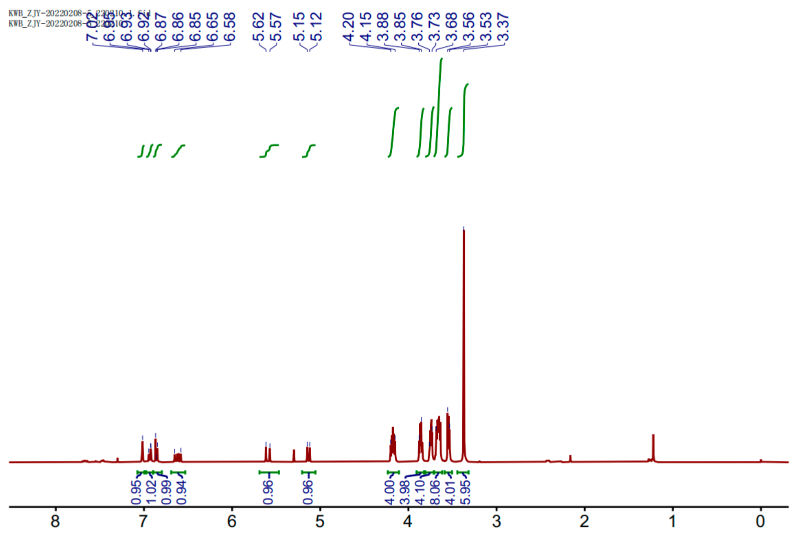

Figure. S1 ^{1}H NMR spectrum of compound **4** in $CDCl_3$ (400 MHz) at 298 K.

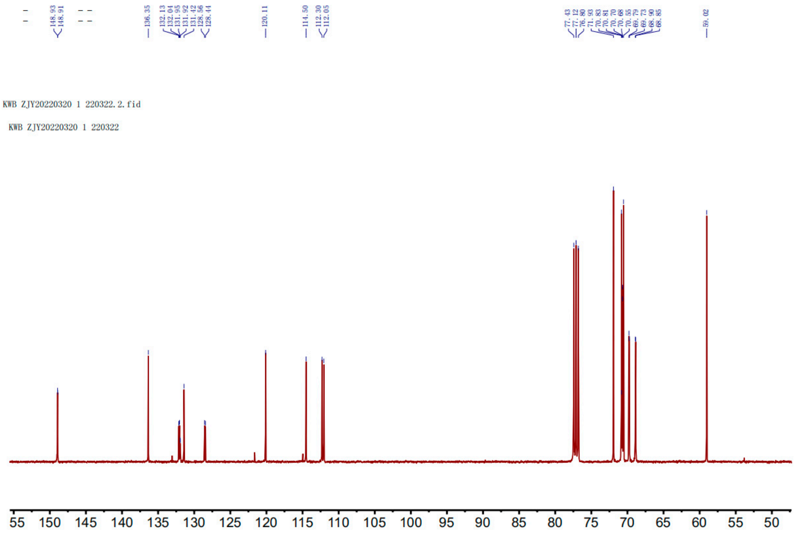

Figure. S2 ^{13}C NMR spectrum of compound **4** in $CDCl_3$ (100 MHz) at 298 K.

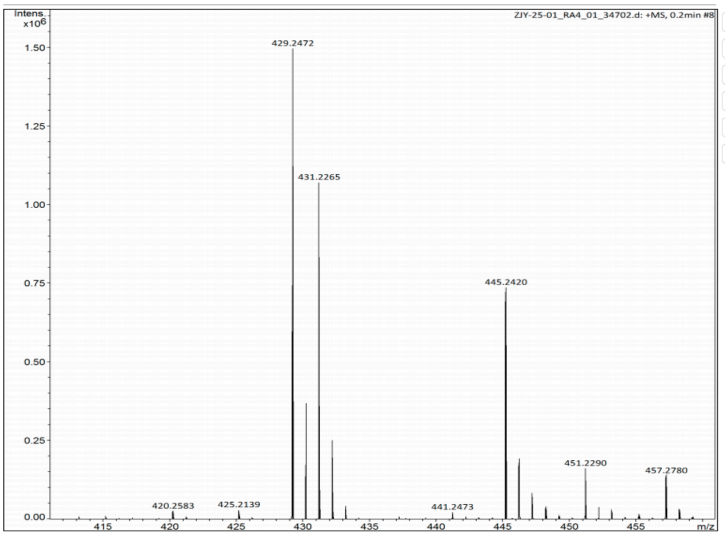

Figure S3. HR-ESI-MS spectra of compound **4**.

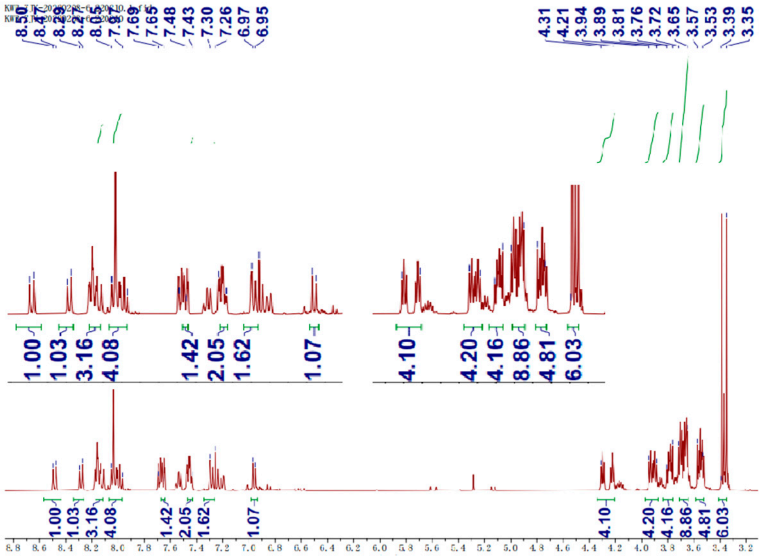

Figure. S4 ^{1}H NMR spectrum of compound **1-H** in $CDCl_3$ (400 MHz) at 298 K.

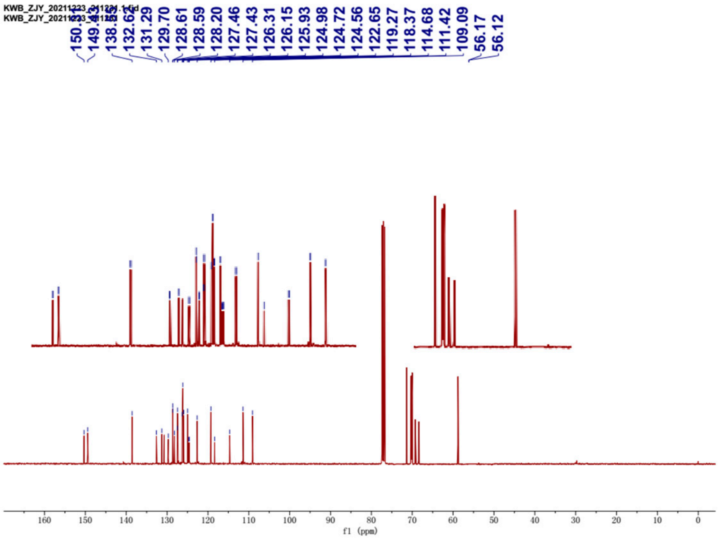

Figure. S5 ^{13}C NMR spectrum of compound **1-H** in $CDCl_3$ (100 MHz) at 298 K.

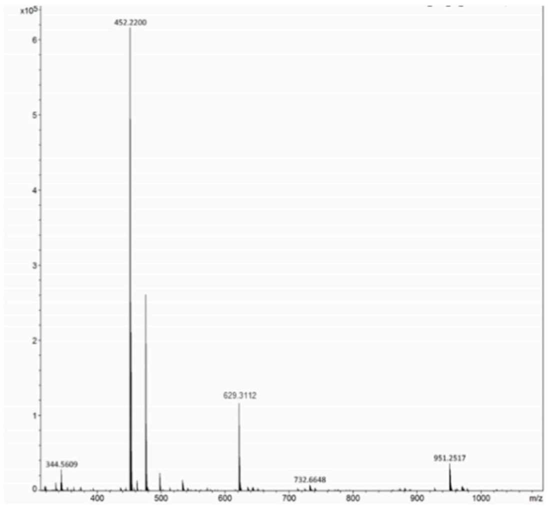

Figure S6. HR-ESI-MS spectra of compound **1-H**.

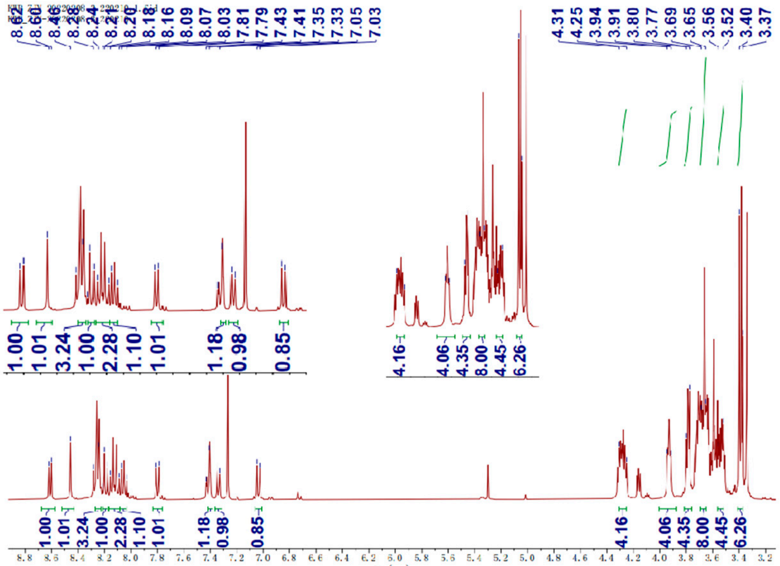

Figure. S7 ^{1}H NMR spectrum of compound **1-CN** in $CDCl_3$ (400 MHz) at 298 K.

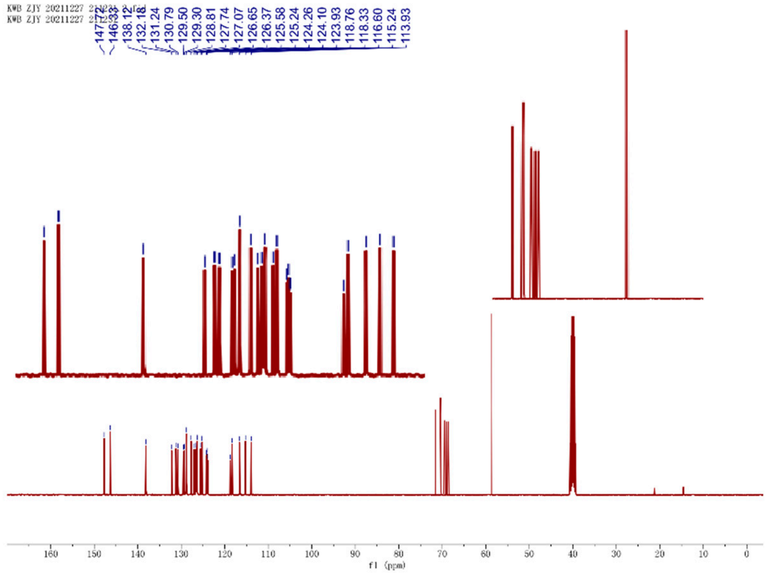

Figure. S8 ^{13}C NMR spectrum of compound **1-CN** in $CDCl_3$ (100 MHz) at 298 K.

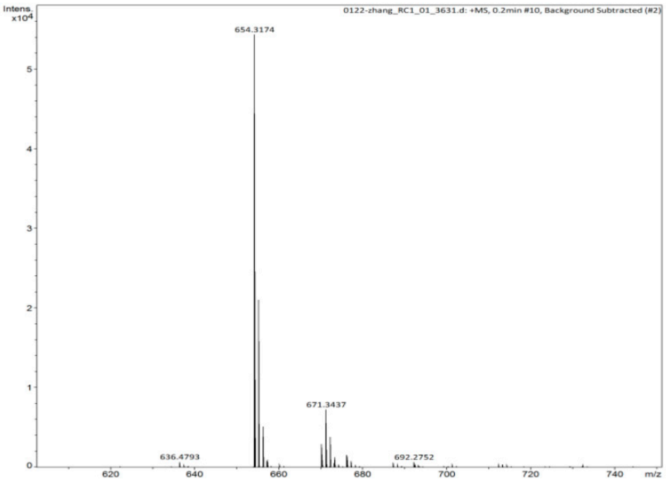

Figure S9. HR-ESI-MS spectra of compound 1-CN.

Table S1: The Photoluminescence Quantum Yield of compound **1-H** and **1-CN**

Compound	State (Solvent)	PLQY (%)
**1-H**	Dispersed (THF)	12.5
**1-H**	Aggregated (H_2O)	3.1
**1-CN**	Dispersed (THF)	8.1
**1-CN**	Aggregated (H_2O)	42.3

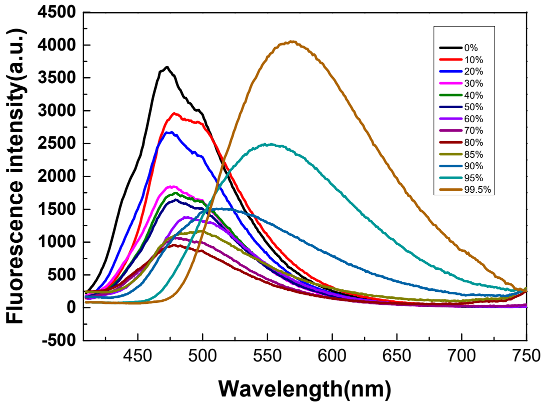

Figure S10. The emission spectra of **1-CN** in binary solvent mixtures of THF-water (λ=365nm, 1 x 10^{-5} M).

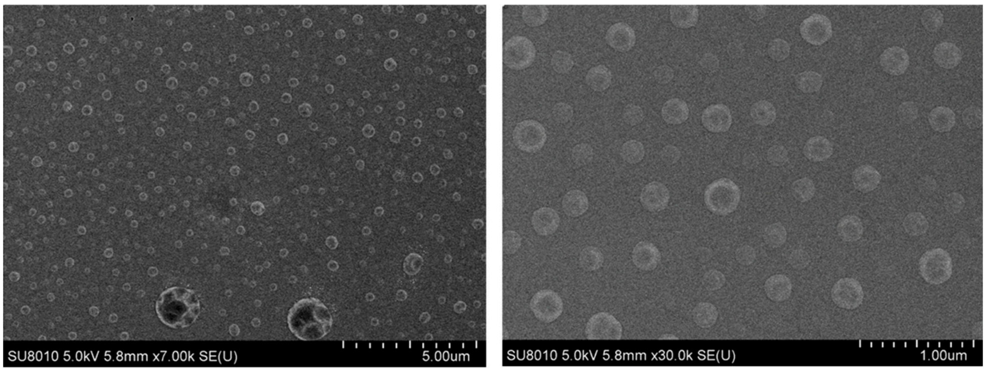

Figure S11. The SEM image of **1-H** prepared in aqueous solution.

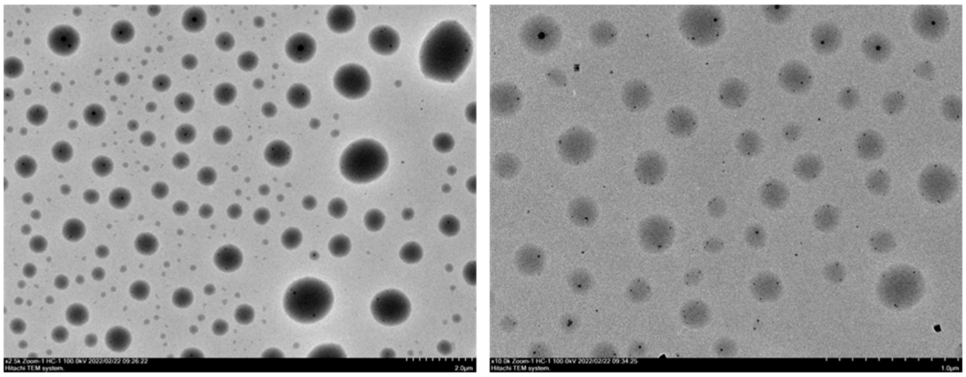

Figure S12. The TEM image of **1-H** prepared in aqueous solution.

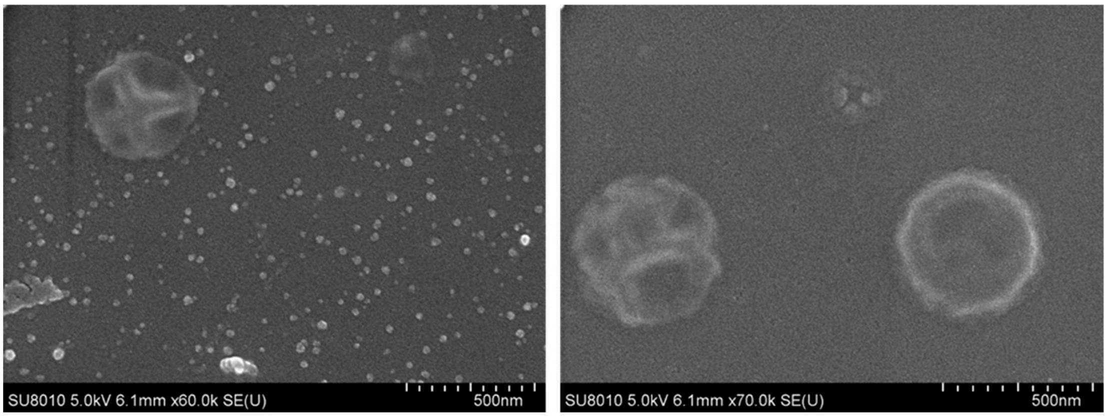

Figure S13. The SEM image of **1-CN** prepared in aqueous solution.

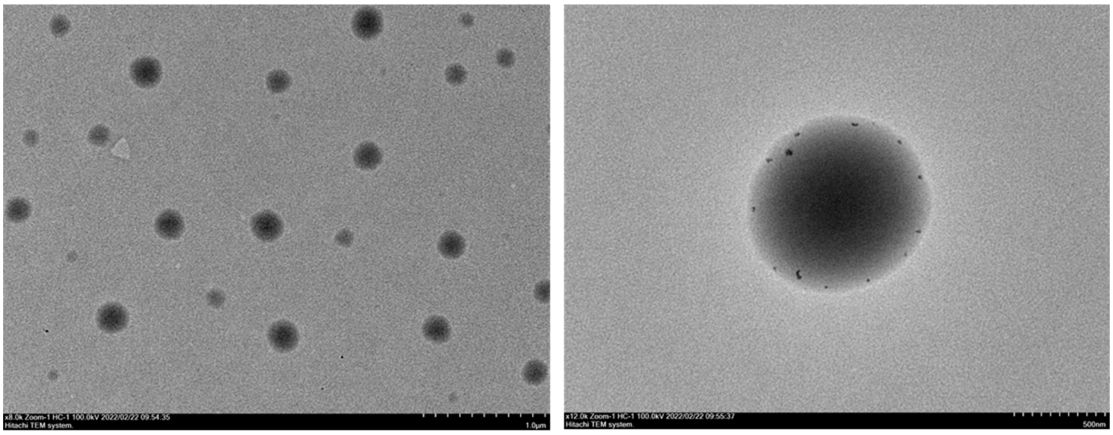

Figure S14. The TEM image of **1-CN** prepared in aqueous solution.

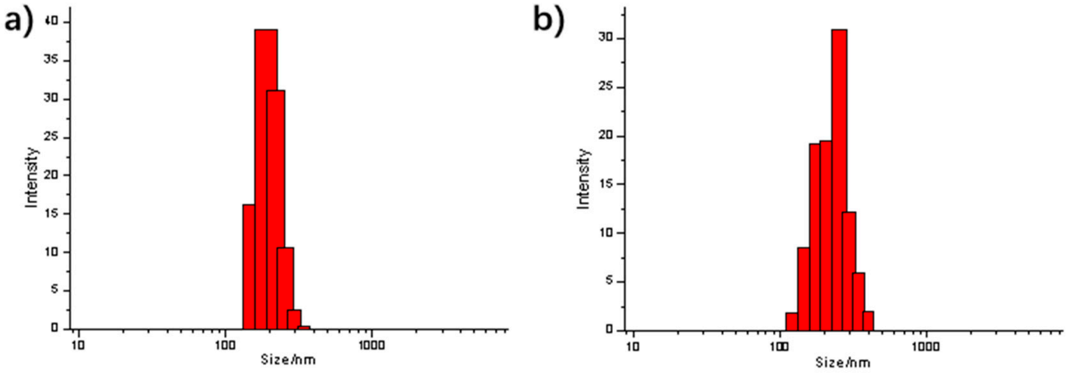

Figure S15. The DLS data of **1-H**(a) and **1-CN** (b) prepared in aqueous solution.
